# Enhanced Cumulative Sum Charts for Monitoring Process Dispersion

**DOI:** 10.1371/journal.pone.0124520

**Published:** 2015-04-22

**Authors:** Mu’azu Ramat Abujiya, Muhammad Riaz, Muhammad Hisyam Lee

**Affiliations:** 1 Department of Mathematical Sciences, Faculty of Science, Universiti Teknologi Malaysia, 81310 UTM Skudai, Johor, Malaysia; 2 Department of Mathematics and Statistics, King Fahd University of Petroleum and Minerals, Dhahran 31261, Saudi Arabia; 3 Preparatory Year Mathematics Program, King Fahd University of Petroleum and Minerals, Dhahran 31261, Saudi Arabia; National Institute of Environmental and Health Sciences, UNITED STATES

## Abstract

The cumulative sum (CUSUM) control chart is widely used in industry for the detection of small and moderate shifts in process location and dispersion. For efficient monitoring of process variability, we present several CUSUM control charts for monitoring changes in standard deviation of a normal process. The newly developed control charts based on well-structured sampling techniques - extreme ranked set sampling, extreme double ranked set sampling and double extreme ranked set sampling, have significantly enhanced CUSUM chart ability to detect a wide range of shifts in process variability. The relative performances of the proposed CUSUM scale charts are evaluated in terms of the average run length (ARL) and standard deviation of run length, for point shift in variability. Moreover, for overall performance, we implore the use of the average ratio ARL and average extra quadratic loss. A comparison of the proposed CUSUM control charts with the classical CUSUM *R* chart, the classical CUSUM *S* chart, the fast initial response (FIR) CUSUM *R* chart, the FIR CUSUM *S* chart, the ranked set sampling (RSS) based CUSUM *R* chart and the RSS based CUSUM *S* chart, among others, are presented. An illustrative example using real dataset is given to demonstrate the practicability of the application of the proposed schemes.

## Introduction

Cumulative sum (CUSUM) control charts have received a great deal of attention in modern industries to monitor unusual variations in manufacturing and service processes. There is a CUSUM location chart for monitoring changes in process mean and CUSUM scale or dispersion chart for monitoring changes in process variability. Many authors have contributed to the theory of the CUSUM charts with emphasis on location charts. However, in reality, monitoring changes in process variability is equally important [1]. An increase in process variance could lead to increase in the number of defective items, and a decrease in process variability made more items closer to the target value resulting in an improved process capability [2]. Scale or dispersion charts are also important in the interpretation of location charts, as the process standard deviation is assumed constant [2].

A number of researchers with the pioneering work of Page in 1963 have studied the use of the CUSUM control chart to monitor process variability. Page [3] studied a CUSUM chart based on subgroup range to monitor increases in the process dispersion. Tuprah [4] evaluated the performance of a CUSUM chart based on subgroup standard deviation. Chang and Gan [5] studied CUSUM chart based of logarithmic transformation of subgroup variance. Acosta-Mejia et al. [2] examined and compared the performance of several CUSUM charts for monitoring process dispersion. Some recent contributions to the advancement of CUSUM chart for monitoring process dispersion include [6, 7] and references therein.

Ranked set sampling (RSS) is a statistical technique for data collection that is currently gaining popularity in the quality control chart. McIntyre [8] introduced the scheme while Takahashi and Wakimoto [9] developed its mathematical theory. RSS has been used to enhance the performance of Shewhart $\overset{-}{x}$ charts for monitoring the process mean [10, 11, 12]. Recently, the RSS methodology has found its way into the memory control charts such as the exponentially weighted moving average (EWMA) and the CUSUM [13, 14, 15] among others. It is well known that these memory charts are more efficient in the detection of small and moderate process shifts than the traditional Shewhart-type charts.

The RSS scheme employs judgment ordering and utilizes extra information from each specific unit to obtain actual samples. Hence, it provides true representative samples about the target population when compared to the traditional simple random sampling (SRS) for the same number of sample observations. In literature, most of the RSS applications in control charting focus on the location chart for monitoring the process mean. Monitoring the process variance based on ranked data have received less attention and is the focus of this paper. In this article, new CUSUM control charts based on extreme RSS, extreme double RSS and double extreme RSS are proposed to further enhance the overall performance of a CUSUM scale chart at detecting a wide range of shifts in process variability.

The rest of the article is organized as follows: Section 2 introduces the RSS method and some of its extreme variations. In Section 3, we present the design structure for the proposed CUSUM chart using the extreme RSS scheme and variants. In Section 4, we discuss the computations of average run length (ARL) and average extra quadratic loss (AEQL) for the proposed charts via Monte Carlo simulation. A comparison of several control charts for monitoring changes in the process dispersion is given in Section 5. Some graphic representations are also given to aid comparison. In Section 6, we present a practical application example of the proposed control schemes using a real dataset. Finally, some concluding remarks are given in Section 7.

## RSS Properties and Variants

The method of RSS introduced by McIntyre involves the drawing of *n* simple random samples each of subgroup size *n* units from the target population. Rank the units in each set by visual comparison, some less-expensive method, or using auxiliary variables, with respect to a variable of interest. The smallest ranked unit is selected from the first set; the second smallest ranked unit is selected from the second set and so on until the largest ranked unit is selected from the last set. The cycle may be repeated *r* times to obtain a ranked set sample of *nr* units. It is important to point out that although RSS requires the ranking of *n* by *n* sample units, only *n* observations are actually measured accurately, and hence, makes the comparison with SRS of same size *n* meaningful [16].

Let *X*_*i*1_, *X*_*i*2_, *X*_*i*3_,…, *X*_*in*_, for *i* = 1, 2,…, *n* be *n* independent simple random samples each of size *n* from a continuous distribution with probability density function *f*(*x*) and cumulative distribution function *F*(*x*). The simple random sample estimates of the population range, *R*, mean, *μ*, and variance, *σ* ^2^, are respectively given by the sample range *R*_srs_, sample mean ${\overset{-}{X}}_{srs}\;$ and sample variance $S_{srs}^{2}$. Let *X*_rss_ = {*X*_(*i*:*n*)*j*_, *i* = 1, 2,…, *n*, *j* = 1, 2, …, *r*} denotes the *i*th order statistic in *i*th sample of size *n* in *j*th cycle under perfect ranking. Then the sample range based on ranked set sample of subgroup size *n* in *j*th cycle is defined as

$$R_{rssj}=X_{\left(n:n\right)j}-X_{\left(1:n\right)j}$$ (1)

with ${\overset{-}{R}}_{rss}\;=\;\left(1/r\right)\sum_{j\;=\;1}^{r}R_{rssj}$. In an analogous to classical SRS, we define the mean and the standard deviation of *R*_*rss*_ as, $E\left(R_{rssj}\right)\;=\;d_{2}^{*rss}\sigma$ and $\sigma \left(R_{rssj}\right)\;=\;d_{3}^{*rss}\sigma$, respectively, where $d_{2}^{*rss}$ and $d_{3}^{*rss}$ are control chart constants that are subgroup size *n* dependants. We have provided the estimates for the values of $d_{2}^{*}$ and $d_{3}^{*}$ for sample sizes of two to ten, via Monte Carlo simulation of 100,000 iterations, with r = 100 based of different sampling schemes (cf. Table 1).

**Table 1:** Control chart constants for *R* chart of normal order statistics based on different sampling schemes.

*n*	$d_{2}^{*}$				$d_{3}^{*}$			
	RSS	ERSS	EDRSS	DERSS	RSS	ERSS	EDRSS	DERSS
2	1.327	1.327	1.416	1.416	0.937	0.937	0.935	0.935
3	1.929	1.929	2.023	2.023	0.907	0.907	0.865	0.865
4	2.305	2.850	2.984	3.532	0.855	0.861	0.787	0.768
5	2.574	3.090	3.216	3.739	0.813	0.819	0.752	0.739
6	2.781	3.640	3.736	4.558	0.781	0.754	0.700	0.651
7	2.949	3.779	3.874	4.675	0.757	0.736	0.683	0.639
8	3.090	4.139	4.213	5.190	0.738	0.695	0.654	0.592
9	3.210	4.236	4.309	5.271	0.722	0.684	0.644	0.586
10	3.315	4.498	4.475	5.639	0.708	0.657	0.629	0.556

For the estimate of population variance based on ranked set samples, Stokes [17] gave a natural estimator define by

$$S_{rss\left(s\right)}^{2}=\frac{1}{rn-1}\sum_{i=1}^{n}\sum_{j=1}^{r}{\left[X_{\left(i:n\right)j}-{\bar{X}}_{rss}\right]}^{2}$$ (2)

where ${\overset{-}{X}}_{rss}\;=\;\left(1/nr\right)\sum_{i\;=\;1}^{n}\sum_{j\;=\;1}^{r}X_{(i:n)j}$ is an unbiased estimator of the population mean, [9]. The expected value of $S_{rss(s)}^{2}$ is $E\left[S_{rss(s)}^{2}\right]\;=\;\sigma^{2}+{\left[n\left(rn-1\right)\right]}^{-1}\sum_{i\;=\;1}^{n}{\left[\mu_{\left(i:n\right)}-\mu \right]}^{2}$, (cf. Stokes [17]), where *μ*_(*i*:*n*)_ is the mean of *i*th order statistic defined by

$$\mu_{\left(i:n\right)}=\int_{-\infty }^{\infty }x\frac{n!}{\left(i-1\right)!\left(n-i\right)!}F^{i-1}\left(x\right){\left[1-F\left(x\right)\right]}^{n-1}f\left(x\right)\;dx.$$ (3)

However, unlike $S_{srs}^{2}$ which is an unbiased estimator of population variance, $S_{rss(s)}^{2}$ is biased. To obtain an estimator, which is unbiased, a modified version of $S_{rss(s)}^{2}$ [16, 18], is expressed as

$$S_{rss}^{2}=\frac{1}{rn-1+\upsilon_{rss}}\sum_{i=1}^{n}\sum_{j=1}^{r}{\left[X_{\left(i:n\right)j}-{\bar{X}}_{rss}\right]}^{2}$$ (4)

where $\upsilon_{rss}\;=\;\left(1/n\right)\sum_{i\;=\;1}^{n}\upsilon_{\left(i:n\right)}$ is a correction constant that depends on sample size *n* and *υ*_(*i*:*n*)_ = [*μ*_(*i*:*n*)_—*μ*]/*σ*. We also define $E\left(S_{rss}\;\right)\;=\;c_{4}^{*rss}\sigma$ and $\sigma \left(S_{rss}\right)\;=\;c_{5}^{*rss}\sigma$, where $c_{4}^{*rss}$ and $c_{5}^{*rss}$ are the *S* chart constants that depends on subgroup size *n*. We have as well provided the estimates via Monte Carlo simulation of 10^7^ iterations with single cycle, for the values of $c_{4}^{*}$ and $c_{5}^{*}$ based on sample sizes of n = 2 to 10 using different sampling strategies in Table 2.

**Table 2:** Control chart constants for *S* chart of normal order statistics based on different sampling schemes.

*n*	$c_{4}^{*}$				$c_{5}^{*}$			
	RSS	ERSS	EDRSS	DERSS	RSS	ERSS	EDRSS	DERSS
2	0.817	0.817	0.834	0.834	0.577	0.577	0.551	0.551
3	0.906	0.906	0.921	0.921	0.422	0.422	0.389	0.389
4	0.941	0.958	0.969	0.979	0.339	0.288	0.247	0.204
5	0.958	0.969	0.977	0.984	0.286	0.248	0.213	0.180
6	0.969	0.982	0.987	0.993	0.249	0.191	0.161	0.119
7	0.975	0.985	0.989	0.994	0.220	0.174	0.148	0.112
8	0.980	0.990	0.993	0.996	0.199	0.144	0.121	0.085
9	0.983	0.991	0.992	0.997	0.182	0.136	0.114	0.082
10	0.986	0.993	0.995	0.998	0.167	0.117	0.098	0.067

In RSS scheme, the ranking of units in a subgroup with respect to the variable of interest may be perfect (without ranking errors) or imperfect (with ranking errors or using auxiliary variables). In reality, imperfect ranking may be unavoidable and the accuracy of ranking depends on the linear relationship between the variable of interest (*X*) and its auxiliary variable (*Y*). Suppose that (*X*, *Y*) has a bivariate normal distribution and assume the regression of the main variable of interest *X* on the auxiliary variable *Y* is linear. Then following Stokes [19], *X* = *μ*_x_ + *ρ*_*xy*_ (*σ*_*x*_ / *σ*_*y*_)(*Y*—*μ*_*y*_) + *ε*, where *μ*_*x*_, *μ*_*y*_, *σ*_*x*_ and *σ*_*y*_ are the population means and standard deviations of *X* and *Y*, respectively, and *ρ*_*xy*_ is the correlation between *X* and *Y* variables. The *ε* is the error term with mean 0 and variance $\sigma_{x}^{2}\left(1-\rho_{xy}^{2}\right)$. Let *X*_[*i*:*n*]*j*_ denotes the *i*th judgment order statistic of the variable of interest *X* based on auxiliary variable *Y*, then the above equation can be written as

$$X_{\left[i:n\right]j}=\mu_{x}+\rho_{xy}\frac{\sigma_{x}}{\sigma_{x}}\left[Y_{\left(i:n\right)j}-\mu_{y}\right]+\varepsilon_{ij}$$ (5)

where *Y*_[*i*:*n*]*j*_ is the *i*th order statistic based on perfect ranking in *j*th cycle of subgroup size *n*.

In practice, ranking of quality characteristics of interest of subgroup sizes greater than four may be difficult, [8]. A very important variation of RSS, which is easier to execute and has the potential of reducing errors associated with ranking, most especially in symmetric distributions, is the extreme RSS (ERSS) introduced by [20]. Some contributions to the theory of ERSS can be found in [21, 22, 23, 24]. The procedure of the scheme is summarized as follows:

- Randomly select *n* random samples each of size *n* units from the target population.
- Rank the units within each set by visual inspection or some less-expensive method, with respect to the quality characteristics of interest.
- If the subgroup size is even, select the lowest rank unit from the first *n*/2 samples in a subgroup and the highest rank unit from the second *n*/2 samples.
- For odd subgroup size, measure the median observation from one sample, and select the lowest rank from the first (*n*—1)/2 samples and the highest rank from the other (*n*—1)/2 samples.
- The steps may be repeated *r* times to obtain a sample of *nr* units of ERSS data.

There are also double extensions of the ERSS schemes namely, extreme double RSS (EDRSS) and double ERSS (DERSS) which are more efficient than the RSS and ERSS. The EDRSS is a two-stage procedure in which RSS is selected on the first stage and ERSS on the second stage. The DERSS, on the other hand, is a two-stage ERSS. That is, select ERSS on both the first and second stages [25]. These extreme variations of RSS are very effective in the estimation of population standard deviation *σ* than the RSS and its median variations [26], if the underlying distribution is normal.

## New CUSUM Charts for Process Dispersion

Since the first study of Page [3] in which he applied a CUSUM on a subgroup sample ranges to monitor process variability, many researchers have proposed different enhancements to the CUSUM dispersion chart—for example, see [2, 5, 6, 7]. Most of these studies, however, are based on the assumption that samples are drawn from a production line using the traditional SRS. Recent studies by Muttlak and Al-Sabah [22], Abujiya and Muttlak [11], Al-Sabah [27], Jafari and Mirkamali [28], Abujiya et al. [15], Mehmood et al. [12], Abujiya et al. [29] and Mehmood et al. [30] among others, have shown that the use of a well-structured sampling technique such as RSS in control charting had significantly improved the performance of the charts over the random sampling.

To increase the sensitivity of CUSUM scale charts at detecting wide range of variability, we studied CUSUM charts based on *R* and *S* statistics for monitoring the dispersion of a normal process using ERSS, EDRSS and DERSS. For simplicity, these will be referred to as scheme I, II and III, respectively. Throughout this article, we assume that the in-control mean *μ*_0_ and standard deviation *σ*_0_ are known or can be estimated from the preliminary in-control process samples. Also, let *σ*_1_ **≠** *σ*_0_ be the standard deviation that needs to be detected assuming that the process mean does not change.

### The CUSUM—*R* chart

In this subsection, we give the design structure of CUSUM of the range *R* for monitoring the process depression based on the extreme variations of RSS. Let *R*_*erssj*_, *R*_*edrssj*_ and *R*_*derssj*_ denote the sample ranges of the *j*th cycle based on schemes I, II and III respectively. Then the two one—sided standardized CUSUM statistics for detecting increases and decreases in process variability using scheme I, can be expressed as (cf. [2, 6]):

$$C_{erssj}^{+}=max\left[0,\;\;C_{erssj-1}^{+}+\left(R_{erssj}/\sigma_{0}\right)-k_{erss}\right]$$ (6)

$$C_{erssj}^{-}=max\left[0,\;\;C_{erssj-1}^{-}-\left(R_{erssj}/\sigma_{0}\right)+k_{erss}\right]$$ (7)

where *k*_*erss*_ is called reference value defined by $k_{erss}\;=\;d_{2}^{*erss}\left(1+\tau \right)/2$, with $d_{2}^{*erss}\;=\;\left(1/\sigma_{0}\right)E\left(R_{erssj}\right)$ and *τ* = *σ*_1_/ *σ*_0_ is the shift in process standard deviation. Here, *E*(*R*_*erssj*_) can be estimated by ${\overset{-}{R}}_{erss}\;=\;\left(1/r\right)\sum_{j\;=\;1}^{r}R_{erssj}$. If either $C_{erssj}^{+}$ or $C_{erssj}^{-}$ exceeds the predetermined control limit *h* for the first time, then an out-of-control signal is given and *j* is the run length of the distribution. For a zero head start, we set the starting values $C_{erss\;0}^{+}$ and $C_{erss\;0}^{-}$ equal to zero. The advantage of standardizing CUSUM is that it makes parameters of the chart independent of *σ* [2]. Similarly, for schemes II and III, we considered

$$C_{edrssj}^{+}=max\left[0,\;\;C_{edrssj-1}^{+}+\left(R_{edrssj}/\sigma_{0}\right)-k_{edrss}\right]$$ (8)

$$C_{edrssj}^{-}=max\left[0,\;\;C_{edrssj-1}^{-}-\left(R_{edrssj}/\sigma_{0}\right)+k_{edrss}\right]$$ (9)

and

$$C_{derssj}^{+}=max\left[0,\;\;C_{derssj-1}^{+}+\left(R_{derssj}/\sigma_{0}\right)-k_{derss}\right]$$ (10)

$$C_{derssj}^{-}=max\left[0,\;\;C_{derssj-1}^{-}-\left(R_{derssj}/\sigma_{0}\right)+k_{derss}\right]$$ (11)

where $k_{edrss}\;=\;d_{2}^{*edrss}\left(1+\tau \right)/2,\;k_{derss}\;=\;d_{2}^{*derss}\left(1+\tau \right)/2,\;d_{2}^{*edrss}\;=\;\left(1/\sigma_{0}\right)E\left(R_{edrssj}\right)$ and $d_{2}^{*derss}\;=\;\left(1/\sigma_{0}\right)E\left(R_{derssj}\right)$. Although sample range is the simplest measure of variation, it is not always as efficient as the standard deviation, which is the natural measure of variation, [1]. Therefore, we present the CUSUM of *S* chart in the next section.

### The CUSUM—*S* chart

Let *X*_*erssj*_ denotes the extreme ranked set samples of subgroup size *n* in *j*th cycle. Then the estimator of the population standard deviation can be expressed as

$$S_{erssj}=\sqrt{\frac{1}{n-1+\upsilon_{erss}}\sum_{i=1}^{n}{\left[X_{erssj}-{\bar{X}}_{erssj}\right]}^{2}}$$ (12)

where ${\overset{-}{X}}_{erssj}\;=\;\left(1/n\right)\sum_{i\;=\;1}^{n}X_{erssj}$ and *υ*_*erss*_ is the bias correction constant similar to *υ*_*rss*_ of section 2. The standardized two one—sided CUSUM of standard deviation *S* for monitoring the variation of a process based on scheme I is given by

$$Z_{erssj}^{+}=max\left[0,\;\;Z_{erssj-1}^{+}+\left(S_{erssj}/\sigma_{0}\right)-k_{erss}\right]$$ (13)

$$Z_{erssj}^{-}=max\left[0,\;\;Z_{erssj-1}^{-}-\left(S_{erssj}/\sigma_{0}\right)+k_{erss}\right]$$ (14)

where $Z_{erss\;0}^{+}\;=\;Z_{erss\;0}^{-}\;=\;0,\;k_{erss}\;=\;c_{4}^{*erss}\left(1+\tau \right)/2,\;c_{4}^{*erss}\;=\;\left(1/\sigma_{0}\right)E\left(S_{erss}\right)$ and *τ = σ*_1_/*σ*_0_. In a similar manner, we define the standardized CUSUM statistics for scheme II as

$$Z_{edrssj}^{+}=max\left[0,\;\;Z_{edrssj-1}^{+}+\left(S_{edrssj}/\sigma_{0}\right)-k_{edrss}\right]$$ (15)

$$Z_{edrssj}^{-}=max\left[0,\;\;Z_{edrssj-1}^{-}-\left(S_{edrssj}/\sigma_{0}\right)+k_{edrss}\right]$$ (16)

where $Z_{edrss\;0}^{+}\;=\;Z_{edrss\;0}^{-}\;=\;0,\;S_{edrssj}\;=\;\sqrt{1/\left(n-1+\upsilon_{edrss}\right)\sum_{i\;=\;1}^{n}{\left(X_{edrssj}-{\overset{-}{X}}_{edrssj}\right)}^{2}},\;k_{edrss}\;=\;c_{4}^{*edrss}\left(1+\tau \right)/2$ and $c_{4}^{*edrss}\;=\;\left(1/\sigma_{0}\right)E\left(S_{edrss}\right)$. In addition, for scheme III, we have

$$Z_{derssj}^{+}=max\left[0,\;\;Z_{derssj-1}^{+}+\left(S_{derssj}/\sigma_{0}\right)-k_{derss}\right]$$ (17)

$$Z_{derssj}^{-}=max\left[0,\;\;Z_{derssj-1}^{-}-\left(S_{derssj}/\sigma_{0}\right)+k_{derss}\right]$$ (18)

where $Z_{derss\;0}^{+}\;=\;Z_{derss\;0}^{-}\;=\;0,\;S_{derssj}\;=\;\sqrt{1/\left(n-1+\upsilon_{dress}\right)\sum_{i\;=\;1}^{n}{\left(X_{derssj}-{\overset{-}{X}}_{derssj}\right)}^{2}},\;k_{derss}\;=\;c_{4}^{*derss}\left(1+\tau \right)/2$ and $c_{4}^{*derss}\;=\;\left(1/\sigma_{0}\right)E\left(S_{derss}\right)$. An alarm is given when any of the CUSUM statistics in Eqs (13) to (18) exceeds a control limit for the first time. We now evaluate the performance of the proposed CUSUM charts for monitoring process variability.

## Performance Measure of the New Charts

The statistical performances of control charts are generally evaluated in terms of the ARL, the mean of the run length (RL) distribution. In other words, ARL represents the average number of observations plotted on a control chart until the chart gives an out-of-control signal or produces a false alarm. This performance measure may be computed by integral equation approach originally introduced by Page [31], Markov Chain approach of Brook and Evans [32], or a Monte Carlo simulation approach adopted by Hawkins [33], Chang and Gan [5], Mehmood et al. [12], Haq et al. [14] among others. In this article, we used the Monte Carlo simulation approach through an algorithm developed in FORTRAN.

In addition to the computation of ARL, we have also computed the standard deviation of RL (SDRL) distribution often used by researchers as a supplementary indicator of the effectiveness of a control chart. Usually, control charts with smaller out-of-control ARL (ARL_1_) or SDRL (SDRL_1_) value at more points are considered more efficient than the others. But Reynolds and Stoumbos [34] pointed out that, in terms of ARL_1_ and SDRL_1_, no chart will give better performance than the others for all shift values *τ*. Since our objective is to enhance the performance CUSUM control charts at detecting a wide range of process variability across all shifts rather than at a particular point, following Wu et al. [35] and Wu et al. [36], we used the AEQL.

The AEQLmeasures the overall performance of a control chart across a range of shifts rather than at a particular shift [36]. It is defined by:

$$AEQL\;=\;\frac{1}{\left(\tau_{\text{max}}\;-\;\tau_{\text{min}}\right)}\;\int_{\tau_{\text{min}}}^{\tau_{\text{max}}}\;\tau^{2}\;ARL\;(\tau )\;d\tau .$$ (19)

where ARL(*τ*) is the ARLvalues of a particular control chart at standard deviation shift *τ*. *τ*_max_ and *τ*_min_ are the upper and lower bounds of shifts in process standard deviation, respectively. The AEQL is based on a loss function, which takes all factors that affect the quality cost into account [36]. The AEQL of a CUSUM control chart can be computed by integration over a range of shifts in standard deviation. The smaller the AEQL value of a control chart, the better the overall statistical performance of the chart.

If we assume that the observations are normally distributed with mean μ_0_ and standard deviation *σ*_0_ and that the process is initially in-control and remains in-control until it goes out-of-control with a shift in process standard deviation from *σ*_0_ to *σ*_1_ = *τσ*_0_. The shift in standard deviation can be expressed as τ = *σ*_1_/*σ*_0_. If *τ* = 1, the process is in-control, while an out-of-control process is indicated by *τ* > 1 and *τ* < 1, representing increase and decease in process variability respectively. Furthermore, we assume that the in-control process has mean μ_0_ = 0 and standard deviation *σ*_0_ = 1 without loss of generality. All the proposed charts were designed to have in-control ARL_0_ values of 200 with subgroup size of *n* = 5. Using the right combinations of the chart parameters, *h* and *k*, each chart is design to detect 20%, 30%, 40% and 50% increases and decreases in process standard deviation. We have evaluated the run-length properties using simulations of 100,000 iterations for each shift *τ* in process standard deviation, and the results are summarized in Tables 3–6. Also presented in Tables 3–6, is the overall performance measures in terms of AEQL.

**Table 3:** RL properties of the proposed CUSUM—*R* chart for detecting increases in process standard deviation (*n* = 5, ARL_0_ = 200).

		Scheme I				Scheme II				Scheme III			
	*k*	3.399	3.554	3.708	3.863	3.538	3.698	3.859	4.020	4.113	4.300	4.487	4.674
	*h*	3.749	2.853	2.300	1.916	3.262	2.486	2.007	1.672	2.821	2.106	1.666	1.355
*τ*	*σ*_*opt*_	1.20	1.30	1.40	1.50	1.20	1.30	1.40	1.50	1.20	1.30	1.40	1.50
1.00	ARL	200.16	200.08	200.02	200.35	200.05	200.04	200.06	200.37	200.23	200.41	200.35	200.45
	SDRL	194.97	197.08	198.02	199.18	196.29	198.46	199.03	198.79	197.78	199.46	199.87	200.60
1.10	ARL	28.73	32.02	35.71	39.58	26.44	30.57	35.19	39.90	21.69	25.93	30.71	35.53
	SDRL	23.89	28.97	33.64	38.17	22.19	27.75	33.35	38.44	18.07	23.69	29.19	34.48
1.20	ARL	11.30	11.57	12.43	13.64	9.96	10.53	11.73	13.25	7.81	8.33	9.47	10.94
	SDRL	7.50	8.86	10.42	12.10	6.50	8.08	9.79	11.85	4.95	6.29	7.97	9.80
1.30	ARL	6.76	6.48	6.63	6.95	5.90	5.73	6.02	6.49	4.60	4.49	4.70	5.11
	SDRL	3.77	4.23	4.85	5.52	3.18	3.63	4.40	5.15	2.43	2.80	3.38	4.02
1.40	ARL	4.87	4.49	4.38	4.43	4.21	3.93	3.93	4.06	3.30	3.06	3.05	3.18
	SDRL	2.43	2.60	2.86	3.19	2.02	2.22	2.50	2.84	1.52	1.66	1.87	2.19
1.50	ARL	3.84	3.46	3.30	3.26	3.32	3.03	2.92	2.95	2.60	2.37	2.29	2.29
	SDRL	1.78	1.85	1.96	2.11	1.47	1.56	1.68	1.86	1.10	1.17	1.26	1.39
1.60	ARL	3.19	2.84	2.65	2.59	2.77	2.48	2.36	2.33	2.18	1.95	1.86	1.83
	SDRL	1.39	1.41	1.47	1.56	1.17	1.19	1.26	1.34	0.88	0.90	0.94	1.00
1.70	ARL	2.75	2.42	2.25	2.18	2.39	2.12	2.00	1.94	1.90	1.68	1.59	1.55
	SDRL	1.16	1.16	1.18	1.23	0.96	0.97	1.00	1.04	0.75	0.74	0.75	0.77
1.80	ARL	2.43	2.13	1.97	1.90	2.11	1.87	1.76	1.69	1.69	1.50	1.41	1.37
	SDRL	1.00	0.99	0.99	1.02	0.84	0.83	0.84	0.84	0.66	0.63	0.61	0.61
1.90	ARL	2.19	1.91	1.76	1.70	1.91	1.68	1.57	1.52	1.52	1.36	1.28	1.25
	SDRL	0.89	0.86	0.86	0.86	0.74	0.72	0.71	0.71	0.59	0.54	0.51	0.50
2.00	ARL	2.00	1.75	1.62	1.55	1.75	1.54	1.44	1.39	1.40	1.26	1.20	1.17
	SDRL	0.81	0.77	0.76	0.74	0.68	0.65	0.62	0.61	0.54	0.47	0.43	0.41
	AEQL	1.091	0.982	0.934	0.923	0.951	0.866	0.839	0.839	0.754	0.692	0.676	0.682

**Table 4:** RL properties of the proposed CUSUM—*S* chart for detecting increases in process standard deviation (*n* = 5, ARL_0_ = 200).

		Scheme I				Scheme II				Scheme III			
	*k*	1.066	1.114	1.163	1.211	1.075	1.124	1.172	1.221	1.082	1.132	1.181	1.230
	*h*	1.085	0.816	0.646	0.532	0.857	0.640	0.510	0.417	0.631	0.457	0.354	0.280
*τ*	*σ*_*opt*_	1.20	1.30	1.40	1.50	1.20	1.30	1.40	1.50	1.20	1.30	1.40	1.50
1.00	ARL	200.42	200.42	200.71	200.95	200.12	200.51	200.00	201.00	200.88	201.59	201.55	201.52
	SDRL	195.28	197.59	199.18	199.94	196.18	198.78	198.90	200.71	198.26	200.44	199.83	201.05
1.10	ARL	26.71	29.42	32.76	36.09	23.52	27.30	31.39	35.71	19.00	22.84	27.19	31.24
	SDRL	22.07	26.45	30.62	34.50	19.69	24.96	29.80	34.66	15.86	20.82	25.87	30.36
1.20	ARL	10.47	10.63	11.25	12.19	8.77	9.19	10.14	11.42	6.81	7.15	8.03	9.16
	SDRL	6.91	8.10	9.40	10.80	5.70	6.97	8.44	10.21	4.29	5.38	6.71	8.23
1.30	ARL	6.30	5.96	5.98	6.23	5.21	5.01	5.16	5.56	4.01	3.84	3.99	4.28
	SDRL	3.49	3.85	4.35	4.90	2.80	3.20	3.71	4.39	2.07	2.39	2.83	3.33
1.40	ARL	4.52	4.13	3.99	4.00	3.73	3.44	3.40	3.47	2.89	2.65	2.60	2.67
	SDRL	2.25	2.39	2.58	2.84	1.77	1.92	2.15	2.42	1.34	1.44	1.59	1.81
1.50	ARL	3.58	3.20	3.02	2.96	2.94	2.65	2.55	2.54	2.29	2.05	1.97	1.97
	SDRL	1.65	1.69	1.80	1.92	1.29	1.35	1.46	1.59	0.98	1.01	1.08	1.17
1.60	ARL	2.98	2.63	2.44	2.36	2.45	2.19	2.07	2.03	1.92	1.70	1.62	1.60
	SDRL	1.30	1.31	1.36	1.41	1.02	1.05	1.09	1.15	0.79	0.79	0.79	0.84
1.70	ARL	2.58	2.25	2.08	1.99	2.13	1.88	1.76	1.71	1.67	1.47	1.40	1.38
	SDRL	1.09	1.08	1.10	1.12	0.87	0.86	0.87	0.90	0.67	0.64	0.63	0.64
1.80	ARL	2.28	1.99	1.82	1.75	1.89	1.66	1.55	1.51	1.49	1.33	1.27	1.24
	SDRL	0.94	0.93	0.92	0.93	0.75	0.74	0.72	0.74	0.59	0.53	0.51	0.50
1.90	ARL	2.06	1.79	1.64	1.57	1.71	1.50	1.41	1.37	1.35	1.22	1.17	1.16
	SDRL	0.84	0.82	0.79	0.79	0.68	0.64	0.61	0.61	0.52	0.44	0.41	0.40
2.00	ARL	1.87	1.63	1.51	1.44	1.57	1.38	1.30	1.27	1.25	1.15	1.12	1.10
	SDRL	0.77	0.73	0.70	0.68	0.62	0.56	0.52	0.51	0.46	0.37	0.34	0.32
	AEQL	1.021	0.911	0.861	0.846	0.847	0.767	0.740	0.742	0.667	0.612	0.601	0.607

**Table 5:** RL properties of the proposed CUSUM—*R* chart for detecting decreases in process standard deviation (*n* = 5, ARL_0_ = 200).

		Scheme I				Scheme II				Scheme III			
	*k*	2.781	2.627	2.472	2.318	2.894	2.734	2.573	2.412	3.365	3.178	2.991	2.804
	*h*	3.313	2.345	1.745	1.350	2.658	1.806	1.291	0.958	2.301	1.529	1.083	0.789
*τ*	*σ*_*opt*_	0.80	0.70	0.60	0.50	0.80	0.700	0.60	0.50	0.80	0.70	0.60	0.50
1.00	ARL	200.04	200.35	200.29	200.08	200.04	200.20	200.29	200.63	200.03	200.59	200.57	200.51
	SDRL	193.87	197.42	197.03	197.41	194.31	197.65	198.31	199.08	195.79	199.51	200.25	200.59
0.95	ARL	73.44	87.52	101.07	114.87	63.57	76.35	89.65	102.25	57.20	71.40	86.92	100.07
	SDRL	66.94	83.47	98.54	113.45	58.04	72.49	87.24	100.62	52.51	68.65	84.99	98.08
0.90	ARL	31.88	40.37	51.01	63.97	25.85	32.35	41.09	51.62	21.29	28.03	37.33	48.22
	SDRL	25.50	36.37	48.31	62.09	20.77	29.18	38.65	49.99	16.96	25.50	35.65	47.12
0.85	ARL	16.94	20.35	26.36	34.81	13.51	15.82	20.09	26.43	10.65	12.81	17.15	23.57
	SDRL	11.15	16.29	23.50	32.69	8.80	12.61	17.78	24.87	6.82	10.26	15.45	22.33
0.80	ARL	10.80	11.83	14.52	19.22	8.56	9.09	10.83	14.09	6.68	7.17	8.93	11.88
	SDRL	5.68	8.04	11.64	16.98	4.44	6.09	8.57	12.45	3.38	4.77	7.12	10.65
0.75	ARL	7.79	7.83	8.89	11.21	6.18	5.99	6.64	8.06	4.79	4.66	5.25	6.53
	SDRL	3.33	4.37	6.12	8.99	2.62	3.32	4.56	6.41	1.98	2.54	3.60	5.22
0.70	ARL	6.06	5.72	6.03	7.04	4.81	4.42	4.52	5.09	3.74	3.42	3.52	4.05
	SDRL	2.15	2.61	3.46	4.88	1.69	2.01	2.56	3.52	1.28	1.51	1.99	2.83
0.65	ARL	4.95	4.48	4.45	4.85	3.95	3.47	3.37	3.54	3.07	2.71	2.61	2.76
	SDRL	1.48	1.69	2.11	2.80	1.17	1.32	1.60	2.08	0.89	0.99	1.22	1.62
0.60	ARL	4.19	3.68	3.50	3.61	3.35	2.87	2.68	2.65	2.61	2.25	2.07	2.05
	SDRL	1.07	1.17	1.37	1.73	0.86	0.91	1.06	1.31	0.67	0.69	0.84	1.01
0.55	ARL	3.65	3.13	2.90	2.86	2.93	2.47	2.23	2.12	2.29	1.96	1.72	1.63
	SDRL	0.80	0.87	0.95	1.14	0.68	0.66	0.75	0.91	0.49	0.53	0.64	0.71
0.50	ARL	3.25	2.72	2.48	2.38	2.58	2.18	1.93	1.76	2.09	1.74	1.46	1.35
	SDRL	0.62	0.67	0.68	0.78	0.57	0.47	0.58	0.67	0.32	0.49	0.54	0.53
	AEQL	0.517	0.501	0.535	0.616	0.414	0.394	0.412	0.462	0.328	0.315	0.334	0.384

**Table 6:** RL properties of the proposed CUSUM—*S* chart for detecting decreases in process standard deviation (*n* = 5, ARL_0_ = 200).

		Scheme I				Scheme II				Scheme III			
	*k*	0.872	0.824	0.775	0.727	0.879	0.830	0.782	0.733	0.886	0.836	0.787	0.738
	*h*	0.995	0.711	0.532	0.416	0.729	0.495	0.359	0.269	0.547	0.363	0.259	0.189
*τ*	*σ*_*opt*_	0.80	0.70	0.60	0.50	0.80	0.700	0.60	0.50	0.80	0.70	0.60	0.50
1.00	ARL	200.07	200.81	201.08	201.17	200.22	200.25	200.27	201.56	201.41	200.73	200.00	201.96
	SDRL	194.65	198.08	199.08	199.38	196.65	199.34	199.50	200.39	197.18	198.68	199.28	202.07
0.95	ARL	73.76	88.96	103.61	117.84	62.67	76.37	90.42	105.39	56.48	72.45	88.64	102.86
	SDRL	67.51	85.51	101.09	116.25	57.47	73.34	87.92	103.97	52.11	69.85	86.63	101.41
0.90	ARL	31.56	40.83	52.62	66.85	24.69	32.11	41.78	53.75	20.29	27.93	38.62	50.22
	SDRL	25.43	36.89	50.09	65.09	19.83	29.10	39.61	52.50	16.30	25.41	37.31	49.10
0.85	ARL	16.55	20.34	27.13	36.30	12.61	15.23	19.95	27.42	9.90	12.41	17.33	24.33
	SDRL	10.88	16.46	24.26	34.44	8.25	12.24	17.80	25.95	6.33	10.00	15.74	23.29
0.80	ARL	10.48	11.70	14.74	20.03	7.92	8.59	10.55	14.28	6.16	6.75	8.69	12.00
	SDRL	5.48	7.95	11.96	17.82	4.08	5.78	8.48	12.66	3.06	4.50	6.96	10.85
0.75	ARL	7.53	7.68	8.89	11.45	5.69	5.59	6.33	7.96	4.39	4.33	5.00	6.45
	SDRL	3.19	4.25	6.16	9.21	2.37	3.08	4.37	6.42	1.77	2.32	3.38	5.23
0.70	ARL	5.84	5.58	5.96	7.11	4.41	4.10	4.24	4.91	3.41	3.16	3.30	3.87
	SDRL	2.05	2.51	3.40	4.93	1.52	1.84	2.38	3.40	1.13	1.38	1.84	2.69
0.65	ARL	4.77	4.35	4.38	4.84	3.62	3.20	3.15	3.38	2.82	2.50	2.44	2.60
	SDRL	1.41	1.62	2.04	2.79	1.07	1.20	1.46	1.96	0.80	0.89	1.13	1.50
0.60	ARL	4.04	3.57	3.44	3.57	3.07	2.65	2.50	2.51	2.41	2.08	1.92	1.92
	SDRL	1.02	1.13	1.33	1.68	0.78	0.82	0.97	1.21	0.57	0.64	0.77	0.94
0.55	ARL	3.52	3.04	2.83	2.82	2.67	2.28	2.08	2.00	2.14	1.80	1.59	1.52
	SDRL	0.76	0.83	0.92	1.10	0.63	0.58	0.69	0.83	0.40	0.53	0.60	0.65
0.50	ARL	3.13	2.65	2.43	2.35	2.36	2.05	1.79	1.65	2.00	1.57	1.34	1.26
	SDRL	0.59	0.64	0.65	0.76	0.50	0.43	0.56	0.64	0.27	0.51	0.49	0.46
	AEQL	0.501	0.492	0.534	0.626	0.382	0.371	0.395	0.456	0.306	0.296	0.322	0.379

Based on the results in Tables 3–6, we summarize our findings for the proposed CUSUM scale control charts as follows:

1. There are no significant differences between the in-control ARL_0_ values of the proposed charts and the corresponding SDRL_0_ values (cf. Tables 3–6).
2. As the shifts in standard deviation increase (τ > 1), Tables 3 and 4, or decrease (τ < 1), Tables 5 and 6, both the out-of-control ARL_1_ and SDRL_1_ decrease rapidly, with *h* inversely proportional to *k* (for increases in dispersion) and directly proportional to *k* (for decreases in dispersion).
3. All the proposed charts have greatly enhanced the detection ability of the CUSUM scale chart to a wide range of increases and decrease in the dispersion of a process (cf. Tables 3–6).
4. The proposed schemes are generally more effective at detecting decreases in the process variability (cf. Tables 5 and 6) than for monitoring increases (cf. Tables 3 and 4) in standard deviation, in terms of low, ARL_1_ and SDRL_1_.
5. The out-of-control ARL_1_ and SDRL_1_ of proposed CUSUM of *R* and *S* charts for monitoring increases in the dispersion are, however, smaller than those of decreases in a very small region. Less than fifteen and twenty-five percentage increases for *R* and *S* charts, respectively.
6. The ARL performance for increases in standard deviation indicates that the proposed *R* and *S* charts are particularly very effective at detecting small changes in variation when the parameter *k* is minimum. For larger values of *k*, the charts are more sensitive to moderate and large shifts (cf. Tables 3 and 4) and vice versa for decreases in variation.
7. The overall performance of the proposed charts, in terms of AEQL, indicate significant gains in efficiency of the proposed CUSUM charts, particularly at a detection ability of 40% increase or 30% decrease in standard deviation except for increases in variation based on scheme I (cf. Tables 3–6).
8. The proposed CUSUM of *S* charts appears to outperform all the corresponding proposed CUSUM of *R* charts in terms of ARL_1_, SDRL_1_ and AEQL, particularly in the detection of increases in variability (cf. Tables 3–6).
9. The significant improvement archived, in terms of ARL_1_, SDRL_1_ and AEQL, when monitoring decreases in process variability using CUSUM of *S* chart (cf. Table 5) are practically comparable to CUSUM of *R* chart (cf. Table 6).
10. The proposed CUSUM of *R* and *S* charts based on scheme III appeared to be more effective than the corresponding CUSUM of *R* and *S* charts based on schemes I and II (cf. Tables 3–6).
11. Of all the proposed charts, the CUSUM of *S* charts based on scheme III dominates all other schemes at detecting various changes in process standard deviation for all combinations of *h* and *k*. The proposed scheme I is the least performing (cf. Tables 3–6).
12. In general, all the proposed charts not only maintain the advantages of CUSUM to detect small shifts but have also greatly increased the sensitivity of the charts in detecting very large process shifts in standard deviation (cf. Tables 3–6).

## Comparisons of the Control Charts

In this section, we compare the performance of the proposed CUSUM scale charts with several control charts for monitoring process dispersion. These include: the classical CUSUM of the range *R*, the classical CUSUM of the standard deviation *S*, the FIR CUSUM of *R* chart, the FIR CUSUM of *S* chart, the RSS CUSUM of *R* chart and the RSS CUSUM of *S* chart. The discussions here are based on subgroup size of *n* = 5, and the cases based on other sample sizes have similar conclusions, [5]. Assuming that the underlying distribution is normal and using an in-control ARL_0_ of 500, the control charts are designed to detect 30% increase and decrease in process standard deviation.

The performances of these control charts in monitoring changes in the dispersion are compared in terms of their ARL values. For a better comparison, we also implore the overall performance measures—the AEQL, the Average Ratio of ARL (ARARL) and the Performance Comparison Index (PCI). The ARARLmeasures the overall performance of a control chart across a range of shifts rather than at a particular shift [35]. It is defined by:

$$ARARL\;=\;\frac{1}{\left(\tau_{\text{max}}\;-\;\tau_{\text{min}}\right)}\;\int_{\tau_{\text{min}}}^{\tau_{\text{max}}}\;\frac{ARL\;(\tau )}{ARL\;{\left(\tau \right)}_{benchmark}}d\tau$$ (20)

where ARL(*τ*) and ARL(*τ*)_*benchmark*_ are respectively, the ARL values of a particular control chart and a benchmark control chart at *τ*. The benchmark chart is the one with the smallest ARL_1_. *τ*_max_ and *τ*_min_ are the upper and lower bounds of shifts process standard deviation, respectively. The ARARLof a CUSUM control chart can be computed by numerical methods. Similar to ARARL, the PCI is the ratio between the AEQL of a particular control chart and the AEQLof the benchmark control chart. However, unlike the ARARL, it does not involve integration over a range of shifts. It is defined as:

$$PCI\;=\;\frac{AEQL}{AEQL_{benchmark}}$$ (21)

where the AEQL_benchmark_ is the AEQL value of the best-performing control chart [37]. A control chart with minimal ARARL gives smaller AEQL and PCI values and is subsequently more effective in detecting changes in the process standard deviation. The ARL, ARARL, AEQL and PCI comparison among CUSUM scale charts are presented in Tables 7 and 8 and ordered from left to right based on their detection ability of 30 percent increases or decreases in standard deviation.

**Table 7:** RL comparison among CUSUM charts for monitoring increases in standard deviation (*n* = 5, ARL_0_ = 500, *σ*_*opt*_ = 1.3).

	Classical CUSUM *R* chart	Classical CUSUM *S* chart	RSS CUSUM *R* chart	RSS CUSUM *S* chart	FIR CUSUM *R* chart	FIR CUSUM *S* chart	CUSUM *R* chart Scheme I	CUSUM *S* chart Scheme I	CUSUM *R* chart Scheme II	CUSUM *S* chart Scheme II	CUSUM *R* chart Scheme III	CUSUM *S* chart Scheme III
*k*	2.675	1.081	2.960	1.102	2.675	1.081	3.554	1.114	3.698	1.124	4.300	1.132
*h*	4.929	1.894	4.139	1.350	4.996	1.919	3.576	1.020	3.109	0.799	2.622	0.567
*τ*												
1.00	500.36	500.26	500.14	500.24	500.32	500.28	500.43	501.04	500.14	500.68	500.00	500.02
1.10	69.46	66.43	60.13	53.91	59.25	56.46	49.85	45.36	47.27	41.78	40.20	34.41
1.20	24.13	22.94	19.64	17.33	17.63	16.77	15.18	13.76	13.57	11.73	10.57	8.97
1.30	13.25	12.65	10.61	9.37	8.88	8.45	8.03	7.33	7.03	6.10	5.41	4.59
1.40	9.03	8.59	7.17	6.37	5.80	5.54	5.43	4.97	4.70	4.08	3.61	3.11
1.50	6.87	6.56	5.43	4.83	4.34	4.15	4.13	3.80	3.58	3.12	2.76	2.38
1.60	5.56	5.33	4.40	3.94	3.48	3.35	3.36	3.11	2.90	2.55	2.25	1.95
1.70	4.70	4.49	3.72	3.34	2.95	2.82	2.86	2.64	2.48	2.17	1.93	1.68
1.80	4.09	3.92	3.25	2.91	2.58	2.48	2.50	2.31	2.17	1.91	1.71	1.48
1.90	3.63	3.48	2.89	2.60	2.30	2.21	2.24	2.07	1.95	1.71	1.53	1.34
2.00	3.29	3.15	2.62	2.35	2.09	2.02	2.03	1.89	1.77	1.56	1.40	1.24
2.10	3.00	2.88	2.40	2.16	1.93	1.87	1.86	1.73	1.63	1.45	1.30	1.17
2.20	2.77	2.66	2.23	2.00	1.80	1.74	1.73	1.61	1.52	1.35	1.22	1.12
2.30	2.58	2.48	2.07	1.87	1.70	1.64	1.62	1.52	1.42	1.27	1.16	1.08
2.40	2.41	2.32	1.95	1.76	1.61	1.55	1.53	1.43	1.34	1.21	1.12	1.05
2.50	2.28	2.19	1.84	1.67	1.53	1.49	1.45	1.36	1.28	1.17	1.08	1.04
AEQL	2.733	2.620	2.192	1.970	1.789	1.724	1.701	1.581	1.491	1.325	1.199	1.084
ARARL	2.606	2.494	2.092	1.873	1.723	1.655	1.619	1.496	1.422	1.253	1.131	1.000
PCI	2.521	2.417	2.022	1.817	1.650	1.590	1.569	1.458	1.375	1.222	1.106	1.000

**Table 8:** ARL comparison among CUSUM charts for monitoring decreases in standard deviation (*n* = 5, ARL_0_ = 500, *σ*_*opt*_ = 0.7).

	Classical CUSUM *R* chart	Classical CUSUM *S* chart	RSS CUSUM *R* chart	RSS CUSUM *S* chart	FIR CUSUM *R* chart	FIR CUSUM *S* chart	CUSUM *R* chart Scheme I	CUSUM *S* chart Scheme I	CUSUM *R* chart Scheme II	CUSUM *S* chart Scheme II	CUSUM *R* chart Scheme III	CUSUM *S* chart Scheme III
*k*	1.977	0.799	2.188	0.814	1.977	0.799	2.627	0.824	2.734	0.830	3.178	0.836
*h*	4.002	1.570	3.331	1.135	4.056	1.590	2.918	0.884	2.240	0.614	1.893	0.448
*τ*												
1.00	500.21	499.90	500.08	500.48	500.15	501.19	500.71	500.82	500.50	500.17	500.01	500.21
0.95	205.95	205.82	186.91	189.10	193.74	194.34	175.60	178.61	153.54	154.09	141.24	143.66
0.90	88.59	88.17	75.80	75.89	76.03	75.78	65.51	66.67	51.34	51.20	43.81	43.80
0.85	43.45	43.00	35.00	34.31	33.11	32.75	28.43	28.41	21.81	21.02	17.34	16.69
0.80	24.81	24.38	19.29	18.51	16.77	16.44	15.17	14.93	11.51	10.82	8.95	8.39
0.75	16.39	16.00	12.56	11.91	10.16	9.93	9.67	9.45	7.38	6.82	5.65	5.22
0.70	11.83	11.56	9.11	8.57	6.99	6.81	6.96	6.78	5.32	4.92	4.08	3.75
0.65	9.22	8.98	7.06	6.63	5.29	5.17	5.40	5.25	4.15	3.81	3.18	2.92
0.60	7.57	7.37	5.79	5.43	4.28	4.17	4.42	4.29	3.41	3.13	2.63	2.42
0.55	6.39	6.22	4.89	4.58	3.60	3.51	3.75	3.63	2.89	2.66	2.26	2.11
0.50	5.53	5.38	4.25	3.98	3.11	3.03	3.27	3.17	2.52	2.33	2.05	1.91
0.45	4.88	4.75	3.75	3.52	2.75	2.69	2.92	2.83	2.24	2.11	1.91	1.75
0.40	4.38	4.27	3.37	3.18	2.46	2.41	2.62	2.53	2.07	2.01	1.76	1.51
0.35	3.99	3.88	3.10	2.95	2.24	2.20	2.32	2.24	2.01	1.97	1.51	1.24
0.30	3.65	3.54	2.93	2.75	2.08	2.06	2.09	2.05	1.99	1.89	1.22	1.05
0.25	3.30	3.20	2.75	2.43	2.01	2.01	2.01	2.00	1.97	1.68	1.04	1.00
AEQL	1.257	1.227	0.992	0.935	0.772	0.759	0.765	0.750	0.627	0.588	0.471	0.433
ARARL	2.882	2.815	2.270	2.148	1.788	1.758	1.760	1.726	1.428	1.341	1.085	1.001
PCI	2.903	2.834	2.291	2.159	1.783	1.753	1.767	1.732	1.448	1.358	1.088	1.000

### Comparison with classical CUSUM *R* chart

The classical CUSUM of the range *R* chart introduced by Page [3] outperforms the traditional Shewhart *R* chart as well as the warning line scheme for the detection of changes in process variability [2, 3]. The chart is based on SRS and not only has the ability to give the position of the departure from the target value but also the amount of departures. The ARL performances of the classical one-sided CUSUM *R* chart are presented in column two of Tables 7 and 8. Comparison of the control charts indicates that the proposed schemes have smaller ARL_1_ than the classical CUSUM of *R* chart. This is equally supported by the ARARL, AEQL and PCI values. All the proposed schemes have greater sensitivity of detecting changes in process standard deviation than the classical CUSUM of *R* chart (cf. Tables 7 and 8).

### Comparison with classical CUSUM *S* chart

Acosta et al. [2] considered the classical CUSUM of the standard deviation *S* chart. This control chart provides better sensitivity for detecting small and moderate changes in the variation of a process than the Shewhart *S* chart [3]. The ARL performance of the classical CUSUM *S* chart in column three of Tables 7 and 8 also indicates the superiority of the chart over the classical CUSUM *R* chart but not as effective as the proposed schemes. In other words, all the proposed charts have smaller ARL_1_ than the classical CUSUM *S* chart. Furthermore, the values of ARARL, AEQL and PCI in Tables 7 and 8 also reveal the dominance of the proposed schemes over the classical CUSUM *S* chart.

### Comparison with RSS CUSUM *R* chart

Al-Sabah [27] presented the RSS based CUSUM chart for location to further enhance the detection ability of a standard CUSUM chart for monitoring changes in process mean level. As an enhancement to the CUSUM scale chart, we studied the performance of the CUSUM of *R* chart under RSS. The ARL values of RSS based CUSUM of *R* chart is presented in column four of Tables 7 and 8. Results show that this scheme has smaller ARL_1_ compared to the classical CUSUM of *R* and *S* charts but is less effective than the proposed schemes. From the overall point of view, all the proposed schemes perform significantly better than the RSS CUSUM *R* chart (cf. Tables 7 and 8). For example, the RSS based CUSUM *R* chart for monitoring increases in standard deviation is inferior to its CUSUM *R* chart counterpart based scheme I by 29%, scheme II by 47% and scheme III by over 100% in terms of ARARL and PCI (cf. Table 7).

### Comparison with RSS CUSUM *S* chart

In the same line with the RSS based CUSUM *R* chart, we equally studied the performance of the CUSUM of *S* chart using ranked set samples, and the results obtained for detection of increases and decreases in process standard deviation are displayed in column five of Tables 7 and 8, respectively. From these results, we clearly see that the RSS CUSUM of *S* chart dominates its *R* chart counterpart when monitoring increases in the dispersion. From the computed ARLs however, the two schemes almost perform equally when detecting decreases in variation. Comparison with the proposed schemes indicates that RSS CUSUM of *S* chart has larger ARL_1_ values for all shifts in standard deviation (cf. Tables 7 and 8). Furthermore, the ARARL, AEQL and PCI values in Tables 7 and 8 also reveal the superiority of the proposed schemes over the RSS CUSUM *S* control chart.

### Comparison with FIR CUSUM R chart

Lucas and Crosier [38] suggested and analyzed the effect of the fast initial response (FIR) feature on CUSUM chart. The feature sets the initial value of the upper and lower CUSUM statistic to a specified positive value, usually *h*/2. Here, *h* is the predetermined control limit of a CUSUM chart. The FIR feature generally minimizes the out-of-control ARL_1_ values and as an enhancement, the feature is implemented with the CUSUM of *R* chart. We present the results for monitoring increases and decrease in variability in column six of Tables 7 and 8, respectively. The ARL_1_ values of the FIR CUSUM *R* chart shows that significant improvement is achieved. The chart outperforms the RSS CUSUM *S* chart in all shift points except in a very small region when percentage increase or decrease is less than 30 and 15 respectively. The FIR CUSUM *R* chart has larger ARL_1_ values than the proposed schemes and is substantially less effective at detecting ranges of shifts in process standard deviation than the proposed charts (cf. Tables 7 and 8).

### Comparison with FIR CUSUM *S* chart

Poetrodjojo et al. [39] studied the FIR CUSUM of standard deviation *S* chart using the statistics $V_{i}\;=\;\sqrt{n}{S_{srs}}_{i}\sigma_{1}/\sigma_{0}^{2}$. They used *h*/2 as a head start when controlling increases in process standard deviation. Following Acosta et al. [2], we adopted the statistics *S*_*i*_ = *S*_*srsi*_/*σ*_0_ for the development of FIR CUSUM of *S* chart and the results are given in column seven of Tables 7 and 8. This scheme is certainly more sensitive to departures from target standard deviation than the earlier CUSUM scale charts but not as effective as the proposed schemes, in terms of ARL_1_. Furthermore, the overall performance of the proposed schemes, in terms of ARARL, AEQL and PCI also indicate the superiority of the proposed charts over the FIR CUSUM of *S* chart except for the CUSUM *R* chart of scheme I which are comparable. For example, the ARARL and PCI values of upper one-sided CUSUM chart reveal that the FIR CUSUM of *S* chart is less effective than the corresponding CUSUM of *S* chart based on scheme I by an average 10%, scheme II by an average of 31% and scheme III by average of 62% (cf. Table 7).

### The issue of imperfectness in ranking

**Fig 1:**
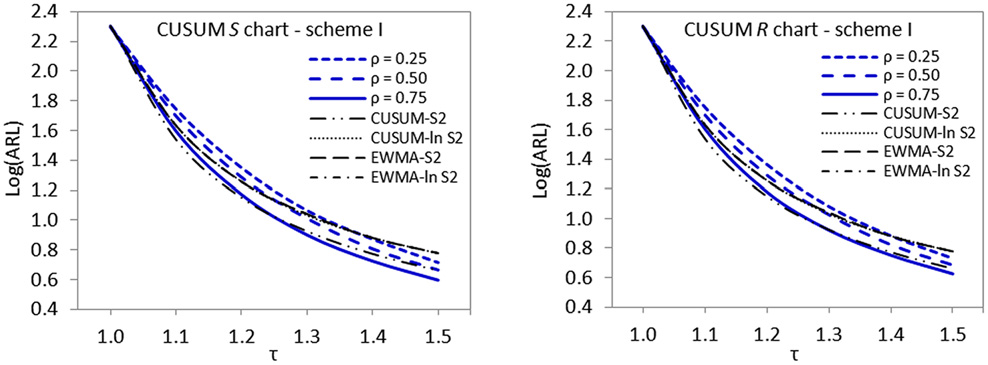
ARL curves for imperfect scheme I versus CUSUM-*S* ^2^, CUSUM-*ln S* ^2^, EWMA-*S* ^2^ and EWMA-*ln S* ^2^ charts for detecting 1.2*σ* when *n* = 5 and *ARL*_0_ = 200.

**Fig 2:**
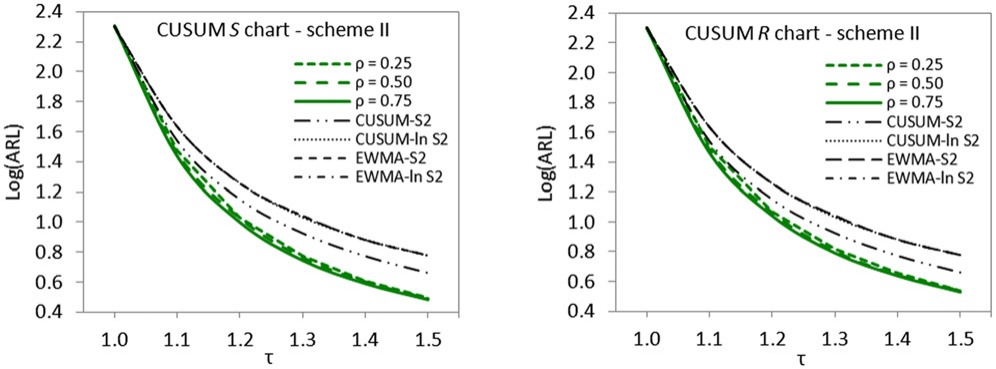
ARL curves for imperfect scheme II versus CUSUM-*S* ^2^, CUSUM-*ln S* ^2^, EWMA-*S* ^2^ and EWMA-*ln S* ^2^ charts for detecting 1.2*σ* when *n* = 5 and *ARL*_0_ = 200.

**Fig 3:**
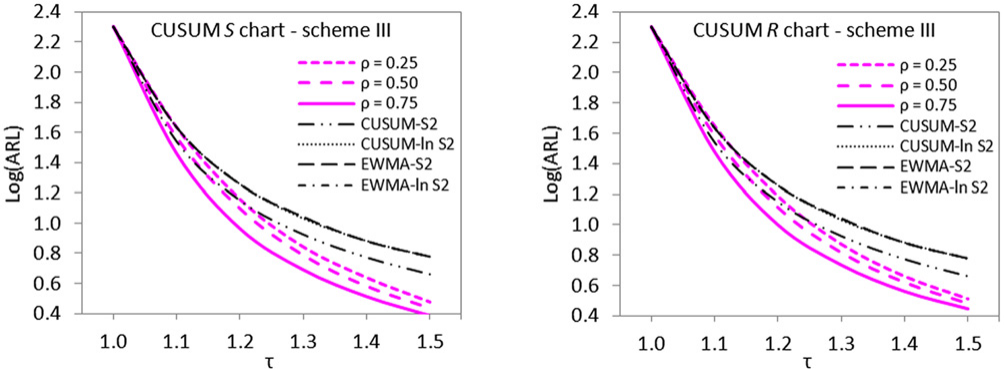
ARL curves for imperfect scheme III versus CUSUM-*S* ^2^, CUSUM-*ln S* ^2^, EWMA-*S* ^2^ and EWMA-*ln S* ^2^ charts for detecting 1.2*σ* when *n* = 5 and *ARL*_0_ = 200.

In practice, ranking of main variable of interest may not always be perfect since the accuracy of the ranking depends on operator judgment. The objective of this sub-section is to investigate the performance of the proposed schemes when ranking of units in each subgroup is not perfect or when ranking of units is carried out with help of an auxiliary variable. We replace the perfect ranking statistics *X*_(*i*:*n*)*j*_ in design structure of the proposed CUSUM scale charts (cf. Section 3) with the imperfect ranking statistics *X*_[*i*:*n*]*j*_, Eq 5. It is well known that perfect ranking and random sampling are special cases of imperfect ranking with correlation coefficients of *ρ*_*xy*_ = 1 and *ρ*_*xy*_ = 0 respectively, [19]. The ARL values of imperfect samples using schemes I, II and III are obtained via Monte Carlo simulation based on data of size *n* = 5 generated from a bivariate standard normal population using *ρ*_*xy*_ = 0.25, 0.5 and 0.75 with an in-control ARL_0_ = 200. The computed ARLs, designed to detect 20% increases in standard deviation, are compared to some existing schemes—the CUSUM of *lnS* ^2^, the CUSUM of the variance *S* ^2^, the EWMA of *lnS* ^2^ and the EWMA of the variance *S* ^2^, using graphical displays of ARL curves (cf. Figs 1–3).

The displayed ARL curves of imperfect cases clearly show that the proposed schemes are not doing badly even in the presence of ranking errors or not very strong correlation between the main quality characteristic of interest and its auxiliary variable. The proposed schemes II and III outperform all the control charts investigated in the comparative studies in the detection of upward shifts in standard deviation (cf. Figs 2 and 3). Scheme I appeared not to be doing very well when *ρ*_*xy*_ < 0.75 but still dominates other control schemes when *ρ*_*xy*_ < 0.75 (cf. Fig 1).

## Practical Applications of the New Schemes

**Fig 4:**
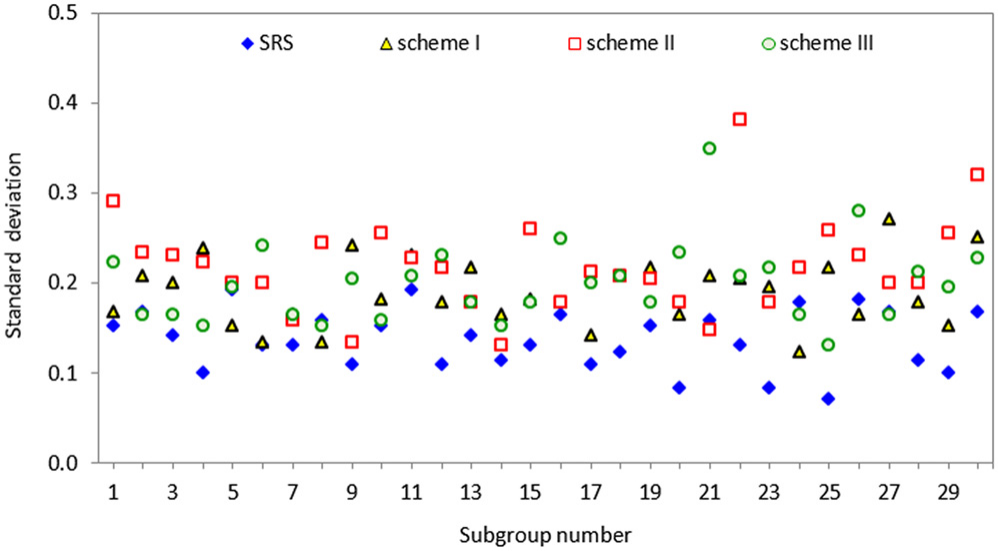
Standard deviations of the thirty re-sampled datasets collected using SRS, schemes I, II and III.

To illustrate the practical application of the proposed CUSUM schemes, we used a real dataset based on the problem of filling bottles on a Pepsi-Cola production line. The original dataset, based on an example in Muttlak and Al-Sabah [10], is from a production line of the Pepsi-Cola production company, Al-Khobar, Saudi Arabia, when the process was in-control. Using the re-sampling approach of Takahasi and Wakimoto [9], thirty random samples each of subgroup size *n* = 5 were collected based on schemes I, II and III, and they reasonably satisfied the normality assumption. Fig 4 displays the standard deviations of the thirty data points for SRS, scheme I, II and III.

**Fig 5:**
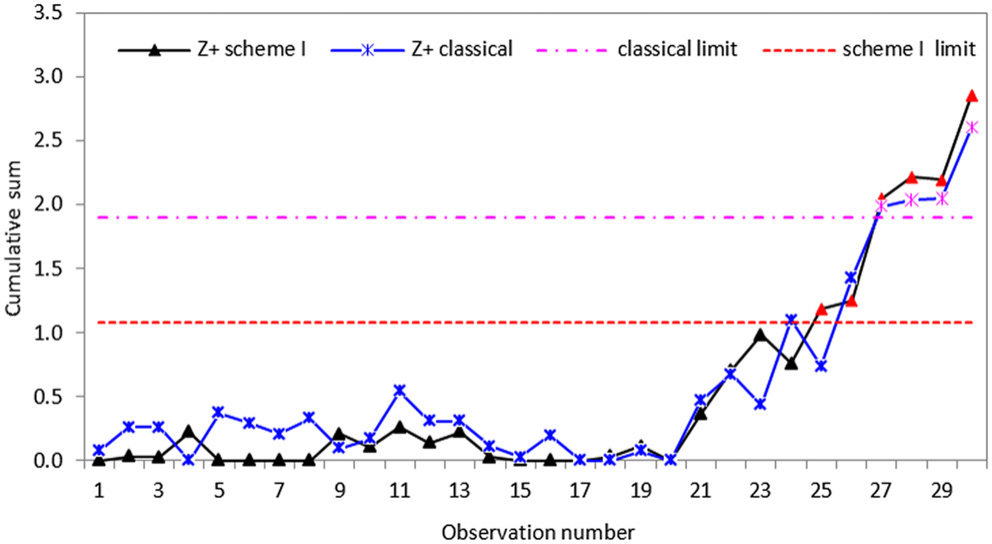
CUSUM *S* control chart using classical and proposed scheme I with *n* = 5.

**Fig 6:**
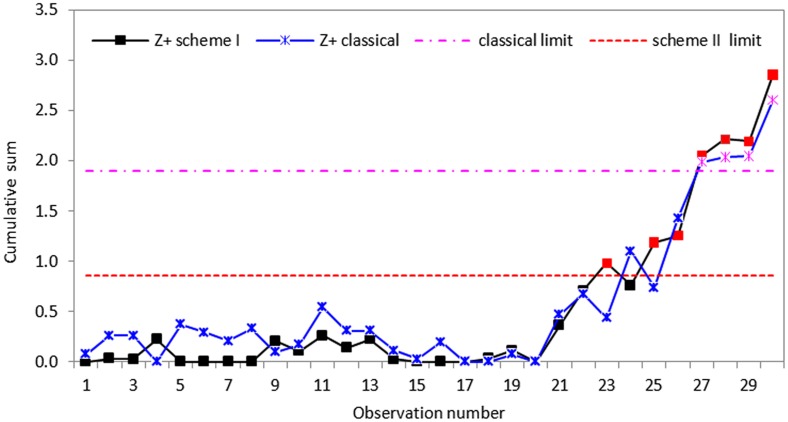
CUSUM *S* control chart using classical and proposed scheme II with *n* = 5.

**Fig 7:**
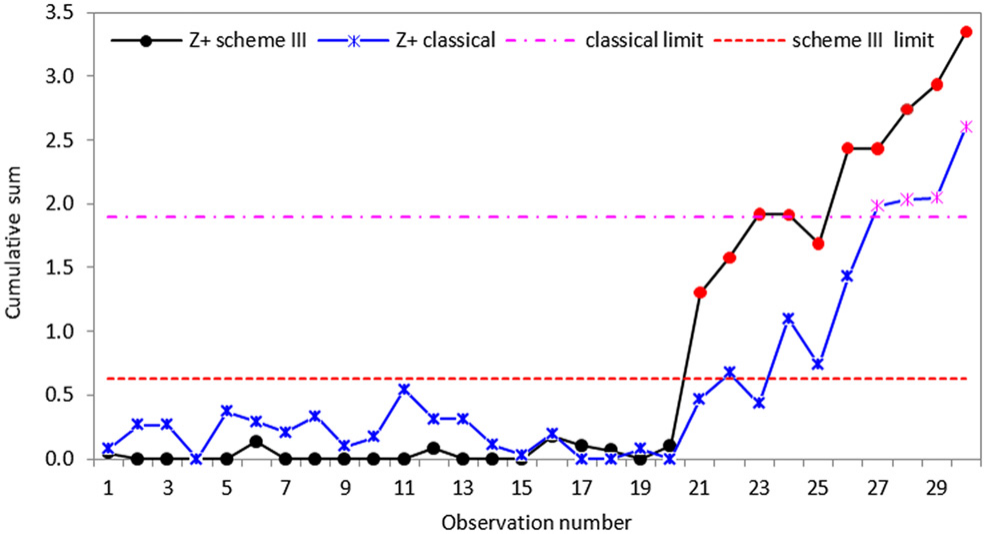
CUSUM *S* control chart using classical and proposed scheme III with *n* = 5.

To measure effectively how the proposed schemes respond to the changes in process standard deviation, some disturbances were introduced to the data by increasing each of the last ten data points by thirty percent to make the process out-of-control. In other words, we assume that the process in-control until the twentieth sample value. Following Hawkins and Zamba [40], we considered increases in standard deviation and compute the statistics of CUSUM *S* control charts for the classical and proposed schemes. Using an in-control ARL_0_ of 200, the control charts were design to detect 20% increases in standard deviation and the graphical representations of the proposed CUSUM *S* charts are displayed in Figs 5–7.

**Fig 8:**
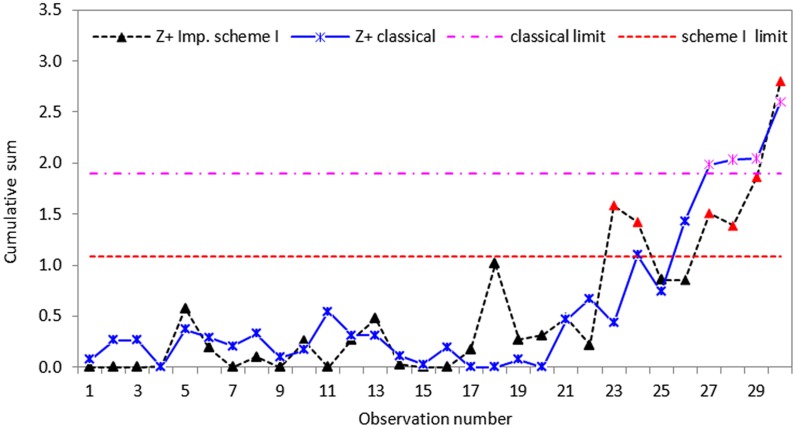
CUSUM *S* control chart using classical and imperfect scheme I with *n* = 5.

**Fig 9:**
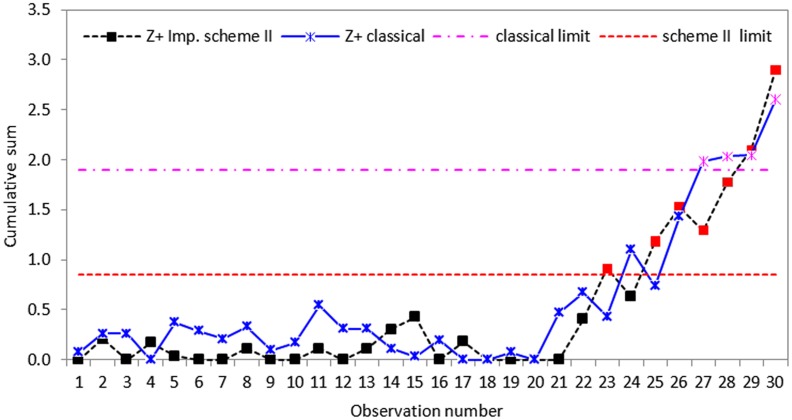
CUSUM *S* control chart using classical and imperfect scheme II with *n* = 5.

**Fig 10:**
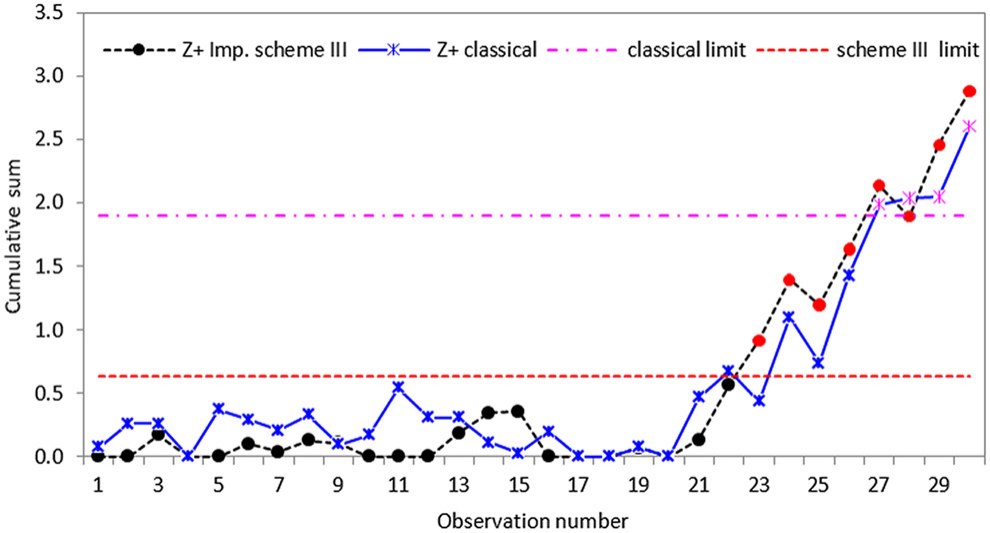
CUSUM *S* control chart using classical and imperfect scheme III with *n* = 5.

To see the effect of imperfect ranking using real dataset, a second set of data is collected using schemes I, II and III with errors in ranking of the bottles with respect to the volume of liquid. In perfect ranking procedure, we placed five randomly selected Pepsi-Cola bottles next to each other, and then ranked them visually with respect to the level of liquid. While in imperfect ranking, a random set of five bottles next to each other in a moving production line is identified and ranked visually as the bottles are moving on the production line. Like the first dataset, thirty random imperfect samples each of size *n* = 5 were obtained using the re-sampling method of Takahasi and Wakimoto [9], from the original imperfect RSS and ERSS dataset in an example in Muttlak and Al-Sabah [10]. We follow the design structure for the first dataset to develop CUSUM *S* charts based on imperfect ranking dataset and the results are displayed in Figs 8–10.

Based on the real dataset used in this application, we observe that the proposed schemes have less variation and are more effective at detecting shifts in process standard deviation than the classical CUSUM *S* chart. Although all the schemes indicate that the process drifts out-of-control from the twenty-first observation, the classical CUSUM *S* chart only gives an out-of-control signal on the twenty-seventh sample, allowing some tiny changes escape detection (cf. Figs 5–7). The perfect cases of propose schemes I, II and III give out-of-control signals at twenty-fifth, twenty-third and twenty-first sample points, respectively. This means that the proposed schemes I, II and III are more effective in detecting tiny changes than the classical scheme with the proposed scheme III giving the correct signal. The imperfect cases given in Figs 8–10, also indicates that the proposed charts are performing better than the classical scheme even with the ranking errors. Conclusions based on CUSUM *R* chart using the real dataset were found to be similar.

## Conclusions

This article proposed some enhanced CUSUM of *R* and *S* control charts based on ERSS, EDRSS and DERSS sampling techniques for monitoring process dispersion. Using Monte Carlo simulations, the performance measures in terms of ARL, SDRL, AEQL, ARARL and PCI, of the proposed CUSUM of *R* and *S* schemes were computed. The performances of the proposed schemes were compared with classical CUSUM *R*, classical CUSUM *S*, FIR CUSUM *R*, FIR CUSUM *S*, RSS CUSUM *R* and RSS CUSUM *S* control charts when standard deviation shifts from its target value. All the proposed schemes were found to be more efficient in detecting a wide range of shifts in the process dispersion than other schemes.

We also examined the performance of the proposed schemes when ranking of the main characteristic of interest is based on auxiliary variable and compared them to CUSUM ln*S* ^2^, CUSUM *S* ^2^, EWMA ln*S* ^2^ and EWMA *S* ^2^. It is observed that even in the presence of ranking errors, the proposed schemes still dominate other procedures. Of all the proposed, the CUSUM *S* chart based on scheme III have the best overall performance and CUSUM *R* chart based on scheme I is the least efficient performance scheme. We have provided real data application of the proposed schemes to demonstrate the practicability of the procedures. In addition, provided are the constant values for *R* and *S* charts of normal order statistics based on different sampling schemes can be used as design aid for the charts. The recommendation is to use the proposed scheme III since it performs better than any other control chart in detecting both increases and decreases in process standard deviation. The scope of this work may be extended to the EWMA charts and multivariate control chart structures.
